# A model predicting the PSP toxic dinoflagellate *Alexandrium minutum* occurrence in the coastal waters of the NW Adriatic Sea

**DOI:** 10.1038/s41598-019-40664-w

**Published:** 2019-03-12

**Authors:** Eleonora Valbi, Fabio Ricci, Samuela Capellacci, Silvia Casabianca, Michele Scardi, Antonella Penna

**Affiliations:** 1 Department of Biomolecular Sciences, University of Urbino, Campus E. Mattei, Via Cà le Suore 2/4, 61029 Urbino (PU), Italy; 2 Department of Biology, University of Rome Tor Vergata, Via della Ricerca Scientifica 1, 00133 Rome, Italy; 3 CoNISMa, Consorzio Interuniversitario per le Scienze del Mare, Pz. Flaminio 9, 00196 Rome, Italy; 4 CNR–IRBIM, Largo Fiera della Pesca 1, 60125 Ancona, Italy

## Abstract

Increased anthropic pressure on the coastal zones of the Mediterranean Sea caused an enrichment in nutrients, promoting microalgal proliferation. Among those organisms, some species, such as the dinoflagellate *Alexandrium minutum*, can produce neurotoxins. Toxic blooms can cause serious impacts to human health, marine environment and economic maritime activities at coastal sites. A mathematical model predicting the presence of *A. minutum* in coastal waters of the NW Adriatic Sea was developed using a Random Forest (RF), which is a Machine Learning technique, trained with molecular data of *A. minutum* occurrence obtained by molecular PCR assay. The model is able to correctly predict more than 80% of the instances in the test data set. Our results showed that predictive models may play a useful role in the study of Harmful Algal Blooms (HAB).

## Introduction

Anthropic pressures, highly increased in recent decades, have strong impact along the coasts of the Mediterranean Sea. Among the consequences, there are eutrophication, a nutrient over-enrichment of coastal waters (especially due to the massive use of fertilizers in agriculture), transport of phytoplankton species via ballast-water vessels and translocation of shellfish stocks^1–4^. In particular, eutrophication is increasing due to increased population, increased use of fertilizers both for terrestrial and marine animal farm practices and increased fossil fuel use^5^. These phenomena can favor a fast proliferation of microalgal species, known as algal bloom^6,7^. Further, climate change seems having effects on the frequency and abundance of algal blooms due to the complex of altered environmental factors^8,9^.

Some microalgal taxa, such as dinoflagellates, can both originate high density biomass proliferation or blooms and produce a variety of toxin compounds that can accumulate along the trophic web through biomagnification process. Such blooms are known as Harmful Algal Blooms (HABs) and they can cause very serious damages to human health and marine organisms^10^. People can be affected either by breathing aerosols^11–13^ or by eating vector species, such as mussels, clams and oysters^14,15^, which can accumulate high concentrations of toxins in their digestive glands. HABs can cause also fish kills or hypoxia or anoxia events due to algal biomass proliferation. Therefore, HABs phenomena, in addition to human health, are also concerned with fishing and aquaculture industry^16–18^.

In recent years, there has been a significant increase of these HABs phenomena worldwide^19–22^, including Mediterranean Sea^23,24^. Therefore, the HAB monitoring programs increased^4^. In the future, the next challenge will be the managing and forecasting of HABs^25^. The mathematical models are shown to be useful tools for this purpose and their use has grown in the last decades. The purpose of these models is to describe^26–29^ or to forecast HABs providing a survey^30–32^, in order to identify environmental, physical and chemical conditions in which the risk of algal blooms is higher and in which it can concentrate efforts, such as sampling frequency to confirm or discharge the predicted bloom. Methods used to build these models are numerical, mathematical, and statistical ones or artificial intelligence techniques, like Artificial Neural Network (ANN)^33,34^ and other Machine Learning (ML) techniques. Recknagel *et al*.^35^ used ANN to predict algal blooms in four freshwater systems. In the northern Adriatic Sea, Volf *et al*.^36^ used predictive model for the phytoplankton abundance. Only a few studies used predictive models for HABs in coastal waters: Asnaghi *et al*.^29^ used a Quantile Regression Forest to predict the concentration of the toxic benthic dinoflagellate *Ostreopsis* cf. *ovata* in Ligurian Sea (North-western Mediterranean) and Kehoe *et al*.^37^ used a Random Forest (RF) to build predictive models of benthic PAR (Photosynthetically Active Radiation) at two sites in Moreton Bay affected by *Lyngbia majuscula* blooms.

In order to develop predictive models, it is crucial to have information about the occurrence of the toxic phytoplankton species. Morphological identification and enumeration of toxic phytoplankton species are usually done by using microscopy methods, which are time-consuming and require taxonomic skills and highly-specialized personnel^38,39^. Moreover, in seawater samples, the target species may be present at very low concentrations, representing only a minor component in the phytoplankton assemblage, and it may risk to remain unnoticed, causing the so-called false negative cases. In addition, morphological identification often stops at genus level failing to discriminate between the various species^40^.

Molecular PCR-based techniques have proven to be very useful tools for qualitative identification of microalgal species in coastal waters^41,42^. PCR methods can quickly detect even limited very low abundance of cells^43^. The process is also far more precise, because species-specific ribosomal DNA regions are amplified by using taxon-specific primers. This reduces the risk of inaccuracy, a fundamental condition for the activation of direct analysis that can enable more accurate diagnosis^44–46^.

In the Mediterranean Sea, most productive areas, due to the nutrient discharged by numerous rivers, are mainly localized at the mouths of big rivers, among them, the Po River in the northern western Adriatic Sea^47,48^. These riverine discharges can generate eutrophication conditions that may lead to bloom events that can be originated by harmful microalgal species or species complex^49^.

The dinoflagellate *Alexandrium minutum* Halim, 1960 is the most widespread toxic species in the western Mediterranean basin^50,51^. This species has been responsible for toxic blooms along the northwestern coast of the Adriatic Sea (Italy) and Ionian Sea, where mussel farms have been contaminated^52,53^. *A. minutum* can produce saxitoxins, GTX1 and 4, that can cause a severe human illness, the Paralytic Shellfish Poisoning (PSP) syndrome^15,54^, the most widespread HAB-related shellfish poisoning illness^55^. In the Mediterranean Sea, the increase in the frequency of toxic *A. minutum* outbreaks and the number of areas affected has coincided with the overdevelopment of coastlines, which increasingly offer confined nutrient enriched waters suitable for microalgal proliferation^3,56^. Generally, nutrient rich waters are trigger for its blooming along coastal waters and the physical structure of mass water is critically for the bloom initiation, avoiding cell dispersion and assuring high nutrient levels. In shallow areas, such as coastal shoreline, beaches, bays, *A. minutum* occurs during spring in coincidence with higher temperature, enhanced rainfall and freshwater inputs, which could be related to the supply of macro- and micronutrients, and with stabilization of the water column^23,57^. Furthermore, despite the dinoflagellates’ preference for settling in confined environments near shore, *A. minutum* has an enormous natural potential for dispersal because of its capacity to grow and produce resting cysts under a wide range of environmental conditions. This feature can be responsible of toxic bloom dispersion^58,59^. Saxitoxin production in *A. minutum* is difficult to be controlled. It is known that the production of STX in some *A. minutum* strains can be influenced by nutritional conditions. In particular, low levels of phosphorus increase it^60–63^. Moreover, grazer-induced toxin production has been shown in *A. minutum* under nutrient replete conditions^64^. Recently, it was found that *A. minutum* responds to pico- to nanomolar concentrations of copepodamides produced by zooplankton with up to a 20-fold increase in production of paralytic shellfish toxins^65^. The *A. minutum* abundance that can determine the toxic levels dangerous for humans and therefore, representing an alert is not known to date, because many variables can influence the contamination of shellfish filter animals (i.e. environmental parameters, cell concentration in the seawater, cellular toxin content); of course, the conditions of pre-bloom and bloom (10^5^–10^6^ cells/L) are supposed to be critical for an alert. But, anyway, the presence of *A. minutum* cells in the seawater can represent a potential for a bloom formation, and therefore, it is crucial both to predict and control its occurrence.

Furthermore, in the Adriatic Sea, the *Alexandrium* species that occur frequently are the toxic *A. minutum* together with no PSP producing *A. mediterraneum, A. pseudogonyaulax, A. tamutum* and *A. taylori*^66^. In some cases, light microscopy examination, which is the traditional method used in the monitoring activity, can’t identify and distinguish exactly the morpho-type species, due to the similarity of morphology. Therefore, it is important having the tools, such as the molecular techniques to identify properly and rapidly the toxic species from the other no PSP producing *Alexandrium* species, and approach analysis to predict its occurrence.

In this study, we developed a model predicting the occurrence of *A. minutum* in the northern western Adriatic coastal water using a Random Forest (RF) (Breiman, 2001), a Machine Learning ensemble technique that combines many Classification Trees (CT). This technique is particularly effective to develop qualitative predictive models, especially when relationships among variables are unknown.

## Methods

### Study sites and sampling

A total of 187 surface seawater samples were collected, monthly, from June 2005 to December 2009 along the transects of the Foglia (43°56′0.55N; 12°56′0.18E) and Metauro (43°50′0.54N; 13°05′0.9E) rivers at 500 m and 3000 m (NW Adriatic Sea) from coastland. Seawater samples were collected at 0.5 m depth using polyethylene bottles, and frozen at −20 °C after filtration (0.45 μm nitrocellulose filters, Millipore, USA) until chemical analyses, or fixed with pure ethanol and stored at +4 °C for molecular determinations.

### Molecular analysis and PCR assay

Molecular PCR analysis was applied both because *A. minutum* is difficult to distinguish from other species within the same genus, as it is characterized by minute details of its thecal plates^67^ and because PCR analysis allows us to be fair more certain about the absence data.

For DNA extraction a volume of 100 mL of surface seawater samples, was filtered through a 25 mm diameter Isopore membrane filters with a pore size of 3.0 µm (Merck Millipore, Billerica, MA, USA) under gentle vacuum to avoid cell disruption. The filters were placed in Eppendorf with 1.0 mL of 95% ethanol and stored at +4 °C. Cells were washed out from the filters with ethanol and collected by centrifugation at 12,500 rpm for 10 min at room temperature. Pellets were kept frozen at −80 °C until molecular analyses. Total genomic DNA was purified from pellets, using DNeasy Plant Mini Kit (Qiagen, Valencia, CA). DNA concentration and integrity were evaluated on 0.8% (w/v) agarose gel using serially diluted λ DNA standards (Thermo Fisher Scientific, Hanover Park, IL, USA) and a gel-doc apparatus (Bio-rad, Hercules, CA, USA).

Species-specific primers for the amplification of *A. minutum* ITS–5.8S rDNA region and PCR conditions were reported in Penna *et al*.^41^. The PCR products were resolved on 1.8% (w/v) agarose 1x TAE buffer gel and were visualized by standard ethidium bromide staining under UV light in a gel-doc apparatus (Bio-rad, Hercules, CA, USA).

### Chemical-physical analysis

Dissolved oxygen, oxygen saturation, salinity, temperature and pH determinations were performed with a CTD probe (Idronaut mod. Ocean Seven 316). The transparency of the seawater column was approached by Secchi depth. Dissolved inorganic nutrients (N-NO_3_, N-NO_2_, N-NH_4_, P-PO_4_ and Si-SiO_2_) and chlorophyll “*a*” were performed spectrophotometrically (Shimadzu mod. UV- 1700) on filtered water samples following the methods of Strickland and Parsons^68^ and APHA AWWA WPCF^69^, respectively. Total phosphorus (TP) was determined on unfiltered water samples according to the method of Valderrama^70^.

### Modelling procedure

Occurrence data (i.e. presence and absence records based on molecular evidence) were associated not only to oceanographic data, but also to other predictive variables, namely day of the year, distance from coastline and three meteorological variables (wind maximum speed, wind direction and cloud cover). Data about the latter variables were retrieved from SYNOP servers.

At first we associated *A. minutum* occurrence data with all the available predictive variables (Table 1) to train RFs. However, at a later stage we also trained a second RF, using only 12 out of the 18 available predictive variables. The reduced data set excluded information about nutrients to make any future use of the model easier, with no need for water sampling and laboratory analysis to determine nutrients concentrations.

**Table 1:** List of environmental parameters used in the training phase.

Variables
Day
Distance from coastline (m)
Wind maximum speed (Km h^−1^)
Wind direction
Cloud cover (okta)
Water transparency (m)
Sea surface temperature (°C)
Salinity (PSU)
Dissolved oxygen (mg L^−1^)
Oxygen saturation (% sat.)
Chlorophyll *a* (μg L^−1^)
pH
N-NO_3_ (μM L^−1^)
N-NO_2_ (μM L^−1^)
N-NH_3_ (μM L^−1^)
P-PO_4_ (μM L^−1^)
Total P (μM L^−1^)
Si-SiO_2_ (μM L^−1^)

Independently of the number of variables used to predict *A. minutum* occurrence, the available records were divided into two different subsets: one third of them was set aside and *a posteriori* used as test set to validate the model. The remaining data were used as a training set, i.e. to provide the information RFs need to grow.

To assign records to the two subsets (training and test), they were first stratified according to *A. minutum* occurrence (presence or absence). Then each resulting subset was sorted according to the day of the year in which samples were collected, as seasonality is a factor that highly influences the presence of *A. minutum*. Then, in each sequence of three records, one was randomly allocated to the test set and the other two to the training set, thus ensuring the homogeneity of the two subsets.

Using both 18 and 12 predictive variables we tested several RFs, each one with different features given by different combinations of three training parameters. These were: the number of trees in the RF (100, 250, 500 or 1000), the number of variables available at each split (3, 4, 5 or 6) and the minimum number of records in each terminal node, i.e. in each “leaf” (1 to 10).

In RFs the overall output is obtained by collecting the output of each tree for each records. In other words, each tree “votes” for one of the possible states of the target variable and the majority wins. In theory, predicting *A. minutum* presence would need 50% + 1 presence predictions from all the trees in the RF. However, especially when the numbers of presence and absence records are not well balanced, the optimal cut-off value for a successful presence prediction can be different. For instance, a RF could be more accurate if it were allowed to predict *A. minutum* presence even when less than 50% of the trees predict that output. In order to optimize the cut-off value to be used instead of 50%, the ROC (Receiver Operating Characteristic) curve^71^ was analyzed to look for the best compromise between true positives and false positives in RF predictions. This way the optimal cut-off value, i.e. the minimum number of presence predictions from the trees in the RF that was needed to issue a presence prediction from the whole RF was found for all the RFs we trained. This procedure was especially necessary because the numbers of presence and absence records were not well balanced in our data set (68 presence and 119 absence records, respectively). As absence records were almost twice as much as those of presence of *A. minutum*, the RF training was slightly biased towards the first case, i.e. to the prediction of absence. Therefore, the optimal cut-off was expected to be smaller than 50% of the votes from the trees, i.e. smaller than 0.5. The ROC curve analysis also provided an AUC (Area Under the Curve) value, that can be regarded as a measure of overall model accuracy. However, in order to select the best model among those we developed with different sets of training parameters, we relied upon the Cohen’s K statistics^72^.

## Results and Discussion

Using all the available predictive variables and different combinations of training parameters (number of trees, number of variables per split and minimum number of records per leaf) we trained 160 RFs. The optimal cut-off value for each RF, i.e. the one that maximized the true positive to false positive ratio, was obtained from the ROC curve analysis. After cut-off optimization, Cohen’s K values were calculated for the test set. They ranged from 0.54 to 0.7, with a median value of 0.64 and, as expected, they tended to be inversely proportional to the minimum number of records per leaf. As the best candidate for optimal predictive performance we selected the best model out of the 160 we trained, i.e. we chose the one with the largest K value. The optimal RF model was the one with 100 trees, 3 predictive variables selected at each split and fully-grown trees, with only a single record in each leaf. The latter criterion, by the way, is the default option in the original implementation of the RF^73^. The optimized cut-off value for that RF was 0.31 and K values were 0.58 for the training set and 0.7 for the test set, while the ROC curve analysis returned a 0.895 value for the training set and a 0.88 AUC value for the test set. The K values relative to the test set indicated a substantial^74^ to good agreement^75^, whereas the AUC testified an excellent performance of the RF model according to Hosmer and Lemeshow^76^. Table 2 showed the confusion matrices for training and test sets as well as K values and the percentage of Correctly Classified Instances (CCI%), which is another index of the accuracy of the model, even though not as robust as Cohen’s K in the evaluation of unbalanced data set. CCI% ranged from 78.4 to 85.5, respectively for the training and test set.

**Table 2:** Confusion matrices for 18-variables new RF, after cut- off optimization (t = 0.310).

Training set		Predicted values		Test set		Predicted values	
		presence	absence			presence	absence
Observed values	presence	43	3	Observed values:	presence	21	1
	absence	24	55		absence	8	32
	CCI% = 78.4				CCI% = 85.5		
	K = 0.58				K = 0.70		

Nutrient concentrations are often available in coastal monitoring data, but their acquisition requires the collection of water samples and lab analyses, whereas data about all the other predictive variables can be retrieved from meteorological records or from *in situ* measurements obtained from multiparameter probes. Therefore, we trained more RFs using only 12 predictive variables, i.e. excluding nutrient concentrations. As for the previous RF, we tested several combinations of the training parameters, thus obtaining 160 different RFs. After cut-off optimization K values ranged from 0.51 to 0.7, with a median value of 0.62. As for the RF based on 18 predictive variables, K values were mainly influenced by the minimum number of records in RF leaves, although to a larger extent. The model with the best predictive ability was based on 1000 trees, using only 2 candidate variables at each split and fully-grown trees. The optimized cut-off value for the best RF was 0.361 and K values for training and test set were, respectively, 0.59 and 0.7. While the interpretation of K values was exactly the same as in the RF based on 18 predictive variables, the AUC values were 0.891 for the training set and 0.905 for the test set. AUC value for the test set, in this case, was a bit larger than the value for the training set and it was also a bit larger than the value for the test set of the other model, indicating an outstanding accuracy according to Hosmer & Lemeshow^76^. The confusion matrices for both the training and the test set were shown in Table 3, together with K values and CCI%, which, as in the previous case, were higher for the test set.

**Table 3:** Confusion matrices for 12-variables new RF, after cut- off optimization (t = 0.361).

Training set		Predicted values:		Test set		Predicted values:	
		presence	absence			presence	absence
Observed values:	presence	39	7	Observed values:	presence	20	2
	absence	18	61		absence	7	33
CCI% = 80.0				CCI% = 85.5			
K = 0.59				K = 0.70			

**Figure 1:**
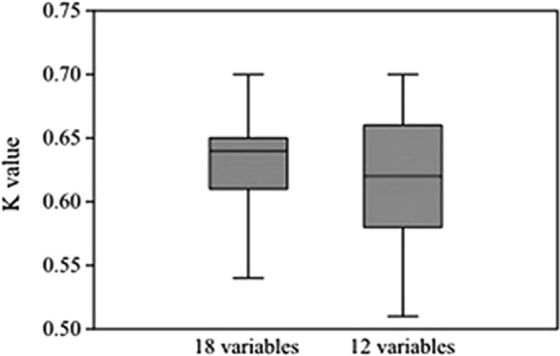
Box plot with K values distribution for all the models tested with different parameters combination. On the left, values of the 18-variables model: minimum value is 0.54, maximum is 0.7. Median value is 0.64. On the right, values of the 12-variables model: minimum value is 0.51, maximum is 0.7 and median value is 0.62.

Comparing the two RFs, the one based on the full set of predictive variables was less dependent than the other one on the optimization of its training parameters, as shown in Fig. 1, where the central quartiles of the K values were narrower than those for the RF based on 12 predictive variables. Moreover, the median K value was larger (0.64 vs. 0.62) in the first case.

However, while using all the predictive variables allowed obtaining less variability depending on the RF training parameters, the best RF model obtained from the reduced set of predictive variables was as good as the best RF model obtained from the full set of predictive variables, if not marginally better (they’re slightly better in the AUC value). Therefore, we have to consider nutrient concentrations as not strictly needed. As obtaining information about nutrients requires additional activities, with larger costs in time and money, we regard the model based on only 12 predictive variables as the best solution to use for making prediction in the future, not only because of its predictive ability, but also because of practical issues.

**Figure 2:**
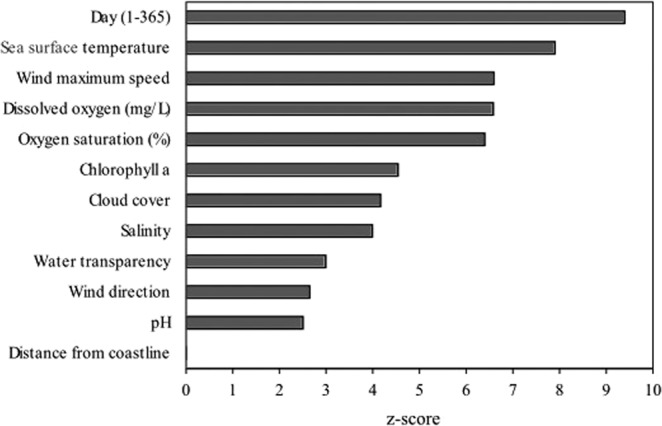
Plot with variable importance for 12-variables RF. The z-scores are obtained by dividing the raw scores by their standard error. All the bars are associated to significant z-scores except the one for distance from coastline, which is non significant and therefore was omitted.

While the main drivers of any model can be identified thanks to sensitivity analysis, an interesting property of the RF algorithm is its ability to support an estimate of the relevance of the role played by each predictive variable. Relative importance of the 12 predictive variables used by the reduced RF model was shown in Fig. 2 as z-scores, computed according to the original algorithm proposed by Breiman^73^.

As we expected, the day of the year, and therefore the period in which samples were collected, is the variable with the largest importance value, and therefore, the most correlated to *A. minutum* presence. In the studied period, the abundance of *A. minutum* was in the range of 10^3^–10^5^ cells/L (data not shown). Sea surface temperature (which is obviously not independent of day of the year, i.e. of season) was the second most important predictive variable, followed by wind maximum speed and oxygen concentration and saturation. Interactions between temperature, wind and oxygen concentration were obvious and certainly modulated by seasonal conditions in favoring *A. minutum* presence. The least important variable, according to the z-score obtained from the RF algorithm, was water pH, which was hardly connected, from a theoretical standpoint, to *A. minutum* presence and possibly affected by relatively large measurement errors.

The main goal of HABs management is to provide early warnings to prevent their impacts on public health and economical activities. Microscope identification of target species is a common procedure, although it requires a great deal of taxonomic expertise, in addition to being time consuming and impractical for processing a large number of samples in a monitoring perspective^38,39,77^.

Recently, HABs phenomena are increasing in the Mediterranean Sea possibly under the influence of the coastal zone overdevelopment^24^. Climate change and global warming are now the main problems that may increase the risk of reaching critical conditions, especially in the Adriatic Sea. The latter is a very shallow sea and one of the most productive regions in the Mediterranean Sea, with nutrient inputs from riverine discharges^78^ and where mussel farms, which play a relevant role in local as well as in Italian mariculture, have already been contaminated^52^.

Results obtained in this study suggest that predictive models may be a valid supplementary tool in HABs management. In fact, they could be very useful to gain important information about those events and to identify the particular conditions in which HABs are more likely to occur, thus supporting the implementation of both new research efforts and activities focused on early reaction, whenever the event should occur.

While our models are already able to correctly predict more than 80% of the real-world instances, the RF approach will allow further improvement as soon as more records about *A. minutum* presence or absence will become available. Moreover, while our model was validated only locally, the same procedure can be applied to other sites or to several sites simultaneously. The ultimate goal, obviously, is a general model, trained and validated in a larger region or across the whole Mediterranean basin.

## Conclusions

Modelling species distribution, both in space and in time, is usually easier when data about species occurrence are not affected by too many error sources. Undetected occurrences are a very common problem among those that may hinder species distribution models and they are more likely to happen than their positive counterpart, i.e. false occurrences, which may depend on species misidentification. While the first source of error depends on sampling design relative to species distribution, the second source only depends on the taxonomical skills supporting the modeler. As for studies on plankton species or assemblages, using molecular methods for species identification solves both problems, because false negatives and false positives are not likely to occur.

As a consequence, even a relatively small data set can support successful modelling if appropriate methods are selected for species identification. This is certainly the case with our study, because species occurrence data were obtained by molecular PCR analyses, which makes us especially confident about absence records. In fact, the latter can be regarded as real absence rather than as misidentification or undetected presence due to very low density of the target species. Confidence in species detection makes us also confident about the accuracy of our model.

This study was carried out for a single species over a relatively restricted area, but the selected approach can be easily applied elsewhere and at any spatial scale. Moreover, its methodological bases allow an easy application to the prediction of a wide range of different target species and this is the reason why RFs are rapidly becoming one of the most widely applied techniques in species-specific distribution modelling.

Our model allows to correctly classify more than 85% cases of presence or absence of *A. minutum*, with values of the K statistics as high as 0.7 for the test set. This result is certainly adequate for supporting an early warning that can be improved.

While the most common goal of any model is to provide accurate predictions, understanding the underlying ecological relationships is a very common secondary or even alternate objective. In our study, the focus was on the prediction of occurrence, but the importance of the predictive variables was assessed by means of the procedure based on the standardized errors in classification of out-of-bag records obtained from RF training. The assessment of the importance of each predictive variable is obviously based on the available data set only, which can be restricted to a limited number of environmental conditions or to limited sequence of events in a more complex time series. From a purely theoretical viewpoint, however, day of the year, sea surface temperature, wind maximum speed and oxygen concentration and saturation are very likely to be associated to conditions in which *A. minutum* is more frequently found. Needless to say, that association is a fact at local space and time scale and just a hypothesis to be tested at larger scale, as often happens when ecological inferences are based on real data sets.

Our model, however, will certainly play a role in predicting, and possibly better understanding, HABs, although it can only help to identify environmental conditions that might favor HABs, not the actual occurrence of those phenomena. As a matter of fact, we still do not have enough data as to try to understand and possibly modelling what triggers a HAB, but our model is certainly able to point out the conditions that are necessary, although not sufficient, to support that type of event. From this viewpoint, machine learning approaches seem particularly promising because they can be easily updated and optimized as soon as new data become available, thus providing useful support to human experts in HAB risk assessment.

## Data Availability

The authors declare the data availability.
